# A Mobile-Based Preventive Intervention for Young, Arabic-Speaking Asylum Seekers During the COVID-19 Pandemic in Germany: Design and Implementation

**DOI:** 10.2196/44551

**Published:** 2023-06-05

**Authors:** Ulrich Frick, Dilan Sipar, Leonie Bücheler, Fabian Haug, Julian Haug, Khalifa Mohammed Almeqbaali, Rüdiger Pryss, Rita Rosner, Hannah Comtesse

**Affiliations:** 1 Research Center, HSD University of Applied Sciences Cologne Germany; 2 Catholic University Eichstätt-Ingolstadt Eichstätt Germany; 3 Institute for Clinical Epidemiology and Biometry, Julius-Maximilians-Universität Würzburg Würzburg Germany; 4 Klinik und Poliklinik für Psychiatrie und Psychotherapie, Bezirksklinikum Regensburg Regensburg Germany

**Keywords:** prevention, COVID-19, refugees, asylum seekers, adolescents, feasibility, behavior planning, vaccination, mobile phone

## Abstract

**Background:**

Most individuals seeking asylum in Germany live in collective housing and are thus exposed to a higher risk of contagion during the COVID-19 pandemic.

**Objective:**

In this study, we aimed to test the feasibility and efficacy of a culture-sensitive approach combining mobile app–based interventions and a face-to-face group intervention to improve knowledge about COVID-19 and promote vaccination readiness among collectively accommodated Arabic-speaking adolescents and young adults.

**Methods:**

We developed a mobile app that consisted of short video clips to explain the biological basis of COVID-19, demonstrate behavior to prevent transmission, and combat misconceptions and myths about vaccination. The explanations were provided in a YouTube-like interview setting by a native Arabic-speaking physician. Elements of gamification (quizzes and rewards for solving the test items) were also used. Consecutive videos and quizzes were presented over an intervention period of 6 weeks, and the group intervention was scheduled as an add-on for half of the participants in week 6. The manual of the group intervention was designed to provide actual behavioral planning based on the health action process approach. Sociodemographic information, mental health status, knowledge about COVID-19, and available vaccines were assessed using questionnaire-based interviews at baseline and after 6 weeks. Interpreters assisted with the interviews in all cases.

**Results:**

Enrollment in the study proved to be very challenging. In addition, owing to tightened contact restrictions, face-to-face group interventions could not be conducted as planned. A total of 88 participants from 8 collective housing institutions were included in the study. A total of 65 participants completed the full-intake interview. Most participants (50/65, 77%) had already been vaccinated at study enrollment. They also claimed to comply with preventive measures to a very high extent (eg, “*always* wearing masks” was indicated by 43/65, 66% of participants), but practicing behavior that was not considered as effective against COVID-19 transmission was also frequently reported as a preventive measure (eg, mouth rinsing). By contrast, factual knowledge of COVID-19 was limited. Preoccupation with the information materials presented in the app steeply declined after study enrollment (eg, 12/61, 20% of participants watched the videos scheduled for week 3). Of the 61 participants, only 18 (30%) participants could be reached for the follow-up interviews. Their COVID-19 knowledge did not increase after the intervention period (*P*=.56).

**Conclusions:**

The results indicated that vaccine uptake was high and seemed to depend on organizational determinants for the target group. The current mobile app–based intervention demonstrated low feasibility, which might have been related to various obstacles faced during the delivery. Therefore, in the case of future pandemics, transmission prevention in a specific target group should rely more on structural aspects rather than sophisticated psychological interventions.

## Introduction

### Background

The COVID-19 pandemic has affected different parts of the population in Germany at different intensities [1]. Asylum seekers and refugees in Germany live to a large degree in collective housing [2]. Asylum seekers in Europe living in such housing conditions have been shown to be exposed to a higher risk of SARS-CoV-2 transmission owing to a much higher contact frequency [3] and longer durations of potentially risky contacts than people living in private flats or houses [4]. In line with this, collectively accommodated asylum seekers were infected with SARS-CoV-2 at a considerably higher attack rate than the general population in Germany [5]. In a review of the prevalence of infectious diseases among refugee groups across the globe, an increased risk for the transmission of more than a dozen other diseases has been shown even before the COVID-19 outbreak [6], and this seems to be attributable to a large degree to the often precarious living situations (eg, unsettled housing conditions or work situations) of refugees in the respective host countries [7].

Knowledge about the COVID-19 disease and its transmission has been shown to be limited among asylum seekers and refugees [8-10]. For example, in a study with Arabic- and Farsi-speaking adult refugees in Germany, the refugee groups displayed significantly less knowledge about COVID-19 and less engagement in preventive behaviors than matched nonrefugee participants [8]. The mitigation of SARS-CoV-2 transmission has been impeded by a simultaneous wave of misinformation on the disease, which has been labeled an “infodemic” [11], spreading mostly via social media. Asylum seekers are a particularly vulnerable group because of their often times insufficient skills in the language of their host country. Thus, they are also at a higher risk for consuming false information [12] about the pandemic and lacking valid information on adequate preventive measures.

Vandormeal et al [13] tested a short, nonverbal, and “culture-agnostic” video to counter social media misinformation about COVID-19 among adolescents and young adults from the United States, United Kingdom, Germany, Spain, and Mexico. They found significantly improved levels of disease knowledge in the video condition compared with an attention control and a do-nothing condition [13]. Similarly, Tjaden et al [14] evaluated a Facebook campaign for COVID-19 vaccine information in a large sample of Arabic-, Turkish-, and Russian-speaking persons in Germany. They showed higher click-through rates for COVID-19 vaccine advertisements than that for average health care–related campaigns on Facebook. Arabic- and Russian-speaking participants showed significantly higher click-through rates when COVID-19 vaccine advertisements were displayed in Arabic and Russian compared with the same advertisements presented only in German. Moreover, a review of smartphone-delivered mental health interventions for asylum seekers and refugees included 12 interventions, of which 3 were specially tailored to adolescent and young refugees [15]. The included interventions varied with regard to the degree of guidance, ranging from unguided (ie, no personal contact or individualized feedback) to guided conditions (ie, different amounts of personal support) [16]. In addition, dropout rates varied widely, ranging from 3% to 80%. Overall, the review showed that participants were largely satisfied with the interventions, indicating that such mobile app–based interventions could be feasible for young asylum seekers.

It is against this background that the Deutsche Forschungsgemeinschaft initiated a research program dedicated to the “prevention of disease transmission in specific social settings and subgroups of the population” on December 14, 2020 [17]. At this time, studies proving the efficacy of messenger RNA vaccines (from Pfizer and BioNTech) were still in assessment, and conditional authorization was announced by the European Medical Association not earlier than December 21.

### Objectives

We proposed the COVID apps for young adults for preventing transmission and promoting vaccination among refugees (CAYPVAR) study to this research program, and the decision to grant the study was announced on April 27, 2021. As the correction of misinformation and myths about COVID-19 and available vaccines seems to be a critical requirement to promote preventive behavior against the transmission of the disease [18,19], the design of our study focused on culture-dependent knowledge of infectious diseases, moral implications of vaccinating, and the most prevalent misinformation (eg, becoming sick or impotent owing to vaccination) in our target group, namely young Arabic-speaking asylum seekers. The major objectives of this study were to answer the three following questions:

1. Can young asylum seekers in Germany be reached to a reasonable extent to roll out a specific prevention campaign during the COVID-19 pandemic?
2. Can a mobile-based intervention with elements from serious games (the CAYPVAR app), providing information in a culture-sensitive and age-adapted mode of presentation, contribute to a better understanding of disease mechanisms and an increased willingness to be vaccinated?
3. Can the potential positive effects of the CAYPVAR app on actual behavior planning be intensified by a face-to-face group intervention? This intervention was planned as a single session and dedicated to addressing the structural barriers prevailing in collective housing and individual needs to understand the disease spread mechanisms. It should contribute to bridging the intention-behavior gap often observed in prevention trials [20].

## Methods

### Study Design

**Figure 1:**
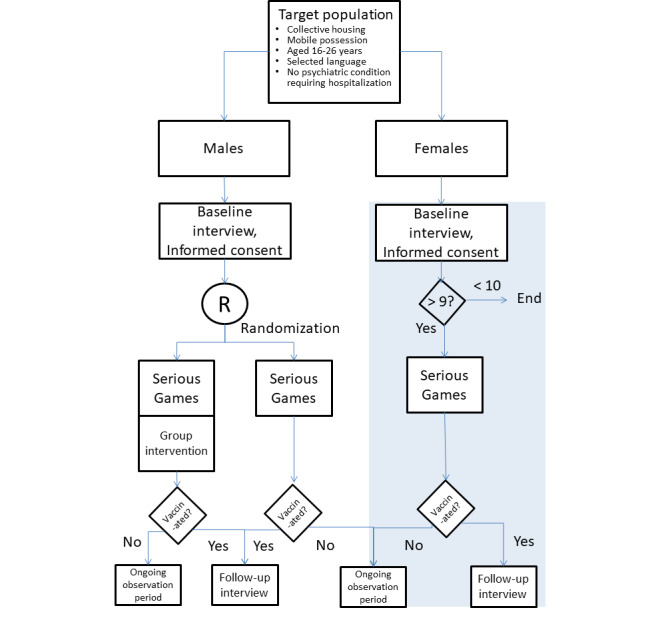
Study design.

The study was planned as a phase II analogous feasibility and quasi “dosage finding” study. The potential efficacy of a mobile app–based informative intervention (the CAYPVAR app) was planned as a pretest-posttest comparison in the first group of participants (group A). A randomly allocated group B was scheduled to receive the CAYPVAR app plus a face-to-face group intervention on behavior planning (Figure 1). This should enable a group comparison of an intensified intervention concept. The CAYPVAR app (group A) was implemented over an intervention period of 6 weeks, and the group intervention was scheduled as an add-on for half of the participants (group B) in week 6.

### Ethics Approval

The study protocol was approved by the Ethics Committee of the HSD University of Applied Sciences (no. 2021/2p, decision made on February 15, 2021) and registered in the German Clinical Trials Registry (study number: DRKS00028825). All participants provided written informed consent before commencing the study. Study data were collected in a pseudonymized manner with different individual codes for the different data sources (ie, interviews, smartphone data, and mobile websites). Personal contact data were never disclosed to team members who analyzed the data. The code list for data merging was only accessible to UF, DS, LB, and HC and was destroyed after the follow-up interviews. Participants were granted free mobile data or Wi-Fi access via prepaid cards or wireless local area network (WLAN) routers installed by the study team during the intervention period. In addition, participants received a one-time voucher of €10 (US $10.9) for continued app use and a voucher of €20 (US $21.7) for participation in the follow-up interview.

### Inclusion Criteria

Inclusion criteria for participants were that they must (1) be aged 16 to 26 years, (2) possess a mobile phone, (3) speak Arabic, (4) be living in a collective housing facility, and (5) not have a psychiatric condition requiring hospitalization. Female asylum seekers aged 16 to 26 years seldom live in collective accommodation, as was known from earlier studies with refugees to Germany undertaken by the working group of the Catholic University Eichstätt-Ingolstadt [21-23]. Therefore, a face-to-face intervention group with female participants was not planned. Female participants would only be tracked regarding their use of the CAYPVAR app if at least 10 female participants could be enrolled.

### Recruitment Strategy

In collective accommodations in Germany, most residents are young male individuals who are waiting for a decision regarding their asylum request or have obtained a temporary residence permit [24]. Thus, their living situation remains unsettled, along with constant uncertainty regarding their possible future residence.

As most asylum requests in Germany have been submitted by Arabic-speaking persons in the last couple of years [2], the largest subpopulation in the collective accommodations approached for recruitment and stated their willingness to participate in this study (altogether 8 institutions) were Arabic-speaking adolescents and young adults (migrating from Algeria, Somalia, Syria, Iraq, Eritrea, Yemen, and Lebanon) [24]. Therefore, all spoken information, video clips, and written materials (eg, quizzes and informed consent forms) were prepared in Arabic. One exception was a short sequence from a classic American science fiction movie (injection scene of a miniaturized submarine into the anterior jugular vein of a Czech scientist, “Fantastic Journey,” 1966 directed by Richard Fleischer), which was embedded into a video on myths and conspiracy theories about vaccines against SARS-CoV-2 (eg, 5G chip implantation) using the unsynchronized English version. During the recruiting visits to the institutions, a professional interpreter was present or one of the longer-established inhabitants not qualifying for the study (mainly because of age) with sufficient or good knowledge of both Arabic and German served as interpreters.

### Intervention Strategy

Most young asylum seekers possess a mobile phone because this has been an important source of information during the flight [25] and is a central mode of connecting with family members and receiving news from home countries [26,27]. Mobile phones of refugees mostly work with prepaid cards as remuneration path. This led us to the idea of installing WLAN routers in the collective accommodation facilities to provide an incentive for study participation, as there was usually no or only unstable internet connection in these facilities.

The intervention period was conceptualized over 6 weeks, and new informative video clips were presented each week (Table 1). Beyond the basic setting of an interview situation in the clips, some elements of gamification were used. For example, sound effects, slapstick-like graphics interchange format scenes of inadequate greeting rituals during the pandemic, links to existing web-based games, or animated cartoons from other educational sources that were embedded in the interview talk were used. All interactive elements strictly respected the privacy of participants. At the end of each week, participants were asked to take a short quiz on the informative videos, and answering these quizzes allowed them to enter the next learning topic. In addition, a link was offered to play a free web-based game (“2020 game”) that recapitulated the world events, including the COVID-19 pandemic, lockdowns, and quarantine, in a jump-and-run format [28]. Finally, a photo and painting competition was held among the participants. As an award for the best pictures on the topic “My life and Corona,” we offered vouchers worth €10 (US $10.9). This should result in a gallery of the CAYPVAR project and thus was intended to sustain participation over the period of 6 weeks by constituting some “sense of being chosen” as a member of the project.

**Table 1:** Overview of the video clips’ content and presentation.

Week and involved persons			Content		Availability
**1**					
	KMA and DS^a^	Biological basis of human cells		Restricted access^b^	
	KMA and DS	Viruses: parasite proliferation and reproduction		Unrestricted	
**2**					
	KMA and DS	SARS-CoV-2: pathogenicity and clinical impact		Unrestricted	
	KMA and DS	SARS-CoV-2: infectiousness over course of illness^c^		Unrestricted	
**3**					
	KMA and DS	SARS-CoV-2: symptoms and differential risk status		Unrestricted	
	KMA and DS	COVID-19: potential long-term effects		Unrestricted	
	KMA solo^d^	Case histories of several of KA’s own patients		Restricted access	
**4**					
	KMA and DS	Preventive measures: social and physical distancing		Unrestricted	
	KMA and DS	Prevention: hand washing		Unrestricted	
	KMA and DS	Prevention: airborne transmission and masks		Unrestricted	
**5**					
	KMA and DS	Vaccination: general mechanism and techniques		Unrestricted	
	KMA and DS	Vaccination: messenger RNA technology and risks^e^		Unrestricted	
	KMA solo	Vaccination: Islamic justification for vaccines		Restricted access	
**6**					
	KMA and DS	Vaccination: herd immunity		Unrestricted	
	KMA and DS	Specific myths on vaccination in Arabic communities		Restricted access	
	KMA solo	Approval of vaccines, fake news among own patients		Restricted access	

^a^KMA and DS: Khalifa Mohammed Almeqbaali and Dilan Sipar in casual clothing; interview situation, YouTube style.

^b^Restricted access: owing to either copyright reasons or privacy protection.

^c^During the construction of videos, only “natural” and Alpha variants were known.

^d^KMA solo: KMA in physician’s overall.

^e^Risks according to evidence in 2021.

### Information Material

To ensure an age-adapted mode of presentation, the videos were scripted as YouTube-style interviews by a female interviewer (DS, subtitled as “psychologist”) asking a male physician (KA, subtitled as “physician”—“Dr. Khalifa”), with both partners sitting in distance on a sofa. Both could be easily recognized as persons from similar cultural backgrounds as the participants. Most interviews contained short sequences such as animated cartoons that explained and visually repeated the verbal information provided by the physician. These sequences stemmed from scientific educational institutions (eg, the FWU Institute for Film and Pictures in Science and Education or the Swiss Office for Public Health). The interviewer asked the questions in German, which were translated into Arabic using the voice-over technique. The answers of the interviewee were freely formulated (but scripted) in simple Arabic language.

In addition, there were videos that presented solo statements of the physician. These clips described real case histories that the physician had treated (eg, for long COVID), drug and vaccine approval procedures, and the safety precautions from his experience as a study physician, and he explained how vaccination from the viewpoint of Islam is justified (ie, protection of other people).

To optimize the video clips for mobile phones, the clips were restricted to a maximum length of 3 minutes, except for a video on vaccination myths, which included a science fiction movie scene from the 1960s (see *Recruitment Strategy* section) and lasted slightly longer than 7 minutes.

The videos were cumulatively made accessible during the 6 weeks of the intervention period, which started individually for each participant from the day of installing the CAYPVAR app on the mobile phone. As shown in Table 1, the sequence of the video clips followed a didactic concept in 6 steps.

A collection of these videos not under specific copyright restrictions is accessible on the web [29].

### Culture-Sensitive Approach

In the early days of 2021, there were no information materials on COVID-19 in Arabic available in Germany [30]. Therefore, a serious and evidence-based source in the mother tongue is necessary for culture-sensitive prevention interventions. Offering this information in an age-adapted simple language and presentation by culturally matched protagonists (KMA and DS), that is, from the same (Arabic) or similar (Kurdish) cultural origin, enables better credibility of the reported facts offered in the video clips [31,32]. Such strategies of using simple language and age-adapted modes of presentation as well as respected trainers have been successful, for example, in adapting trauma-focused preventive interventions for minor refugees in Germany [33].

Typical misunderstandings and fears among the Islamic community had been identified by a Kurdish journalist and blogger in social media formats, such as Facebook or Telegram groups [34]. The journalist advised the study team to script clips on myths and on the manual for group intervention. For example, by approaching the issue of popular misunderstandings or fears on vaccination by an Arabic physician, we expected these fears (eg, “will I become impotent due to the vaccine?”) would be better counteracted than by neutral, distant information.

### IT Infrastructure and Data Collection

Two components were prepared for adequate IT infrastructure. First, a newly connected digital subscriber line connection and a 4G router were installed in collective accommodations where there was no or only an unstable internet connection, with the support of the Bechtle company (as part of an unconditional sponsorship program).

The CAYPVAR app was downloaded by participants from the official Google and Apple app stores using an account provided by the project team during the baseline interview. After the initial log-in, the app downloaded the quiz questions.

The questionnaire-based baseline and follow-up interviews were collected on separate tablets handed to participants (intake interviews) or interpreters (follow-up interviews) for each interview. Interpreters were present throughout the provision of study information and interviews. During the interviews, sociodemographic information (age, education, country of origin, and religious orientation) and vaccination status were obtained. Vaccination readiness (“Do you want to get vaccinated?”) was assessed on a 4-point scale (“yes, absolutely,” “yes, but only with a specific vaccine,” “I am still undecided,” and “no”). The Patient Health Questionnaire-4 (PHQ-4) [35] was used to assess symptoms of depression and anxiety during the past 2 weeks on a 4-point scale (“not at all” to “nearly every day”). The Somatic Symptom Scale-8 (SSS-8) [36] was used to measure somatic complaints during the past week on a 5-point scale (“not at all” to “very much”). The General Self-efficacy Scale was used to measure perceived self-efficacy based on 10 items rated on a 4-point scale [37]. A 9-item questionnaire on attitude toward preventive behaviors against transmission and actual engagement in preventive behaviors was obtained, which included 2 behaviors not feasible to mitigate transmission as retention checks (ie, “healthy food” and “mouth rinsing”; all items are shown in the *Results* section). To assess knowledge of disease mechanisms and transmission paths, a 12-item knowledge test was used, whereas the amount of one’s own knowledge about infectious diseases was assessed using 12 items (all items of the 2 tests are listed in the *Results* section).

Videos of the educational events were loaded (streamed) only when needed. The results of the completed quiz questions and other collected data were cached by the apps and transferred to the server when they were connected on the web. Further details on the IT infrastructure and methods for maintaining participant privacy will be published elsewhere in a separate, more technically oriented paper. In general, our apps follow the technical principles described by Pryss et al [38].

### Add-on Face-to-face Group Intervention

The manual of the group intervention was based on the principles of the health action process approach [39,40]. It is available upon request from HC. The group intervention was designed to foster the translation of prevention intentions into actual preventive behavior by providing planning based on the health action process approach model [41]. It included 2 components: the first one involved action planning of preventive activities of wearing masks, washing hands, and keeping a distance (where, when, how often, how long, and in contact with whom for each activity) and the second one involved coping with planning by identifying possible obstacles (eg, what could prevent you from wearing the mask as planned? Aching ears?) and planning alternative actions (eg, wearing a mask with a headgear).

The setting was prepared as a single group session with 8 participants at maximum lasting for up to 90 minutes. The sessions were to be conducted by DS with the assistance of an interpreter.

### Statistical Considerations

While planning this feasibility study, a vaccine against COVID-19 was not yet approved. However, rumors on side effects of the potential vaccines had already been spread via social media. Therefore, the base rate of the first major study end point, willingness to get vaccinated, was set to a very low number (2%) for power calculation. A pretest-posttest comparison (ie, baseline and after 6 weeks) of increasing the willingness of only half of the included participants would have reached a statistical power of 0.95 in a sample of n=8 (Fisher exact test). All power analyses were performed using G*Power 3.1 (Heinrich-Heine-Universität Düsseldorf).

The second major study end point, knowledge of disease mechanisms, was expected to reach low levels at intake (ie, 4 correct answers in the 12-item knowledge test, with relatively large SD of 4.0). Expecting that processing the contents of the video clips presented via the app would enable us to solve at least 8 (SD 4.0) test items, 12 participants would be needed to reach a power of 0.95.

To detect significant differences between both groups in the follow-up interview (ie, after 6 weeks) with a power of at least 0.90, we expected an increase in the willingness to get vaccinated from 50% (group A) to 80% (group B). This required 47 participants in each group. For an additional increase in disease knowledge (study end point 2) of 3 more correctly solved items, a good statistical power (0.95) could be reached by including 40 participants per group. For analysis of group differences, 2-tailed *t* tests for independent samples or (depending on score distribution) a Kruskal-Wallis test was planned.

## Results

### Access to the Target Population and Enrollment of Participants

During the recruitment period (October 2021 to December 2021), 8 institutions (4 in Bavaria, 4 in Berlin) out of 56 collective housing facilities contacted (thereof 15 in Berlin) agreed to offer their residents the choice to participate in the CAYPVAR study and allowed the study staff to enter the facility. The Bavarian centers were large (up to 1000 inhabitants), whereas the considerably smaller Berlin centers contributed 5 participants at maximum. Therefore, these small 4 institutions in Berlin were treated as 1 common Berlin center for comparison. This reflects the differing legal regulations in Berlin compared with Bavaria during the observation period.

Study enrollment proved difficult in a dynamic pandemic situation and under changing legal regulations. Collective housing institutions, especially in Bavaria, with a greater number of asylum seekers living in one building, were reluctant to allow access to their inhabitants. Some centers even withdrew their willingness to participate in the study because of increased regional incidence rates.

The enrollment of participants began on November 8, 2021. At the start of enrollment, Germany’s second (partial) lockdown was put into effect. During the preceding time, when information material had been prepared, the prevailing virus variant was Alpha (B.1.1.7). This had changed to the Delta variant (B.1.617.2) when the last baseline interview took place (December 16, 2021). The Delta variant had a considerably higher contagiosity than Alpha, causing a higher death toll in susceptible persons. Therefore, collective housing facilities in Bavaria severely tightened contact restrictions, rendering face-to-face group sessions unfeasible. In addition, various housing institutions linked their study acceptance to the precondition that all their inhabitants should profit from the incentives (free or improved WLAN access) of CAYPVAR. Thus, randomization of participants into 2 groups would have been possible only as cluster randomization, covering a very limited number of housing centers. Owing to the difficult enrollment situation in combination with a rapidly changing residential population (eg, short-term relocation of study participants to other regions) and tightened contact restrictions, it was decided to abandon the randomized additional intervention (group B) and to only evaluate the efficacy of the mobile app–based intervention (ie, CAPYVAR app, group A).

**Figure 2:**
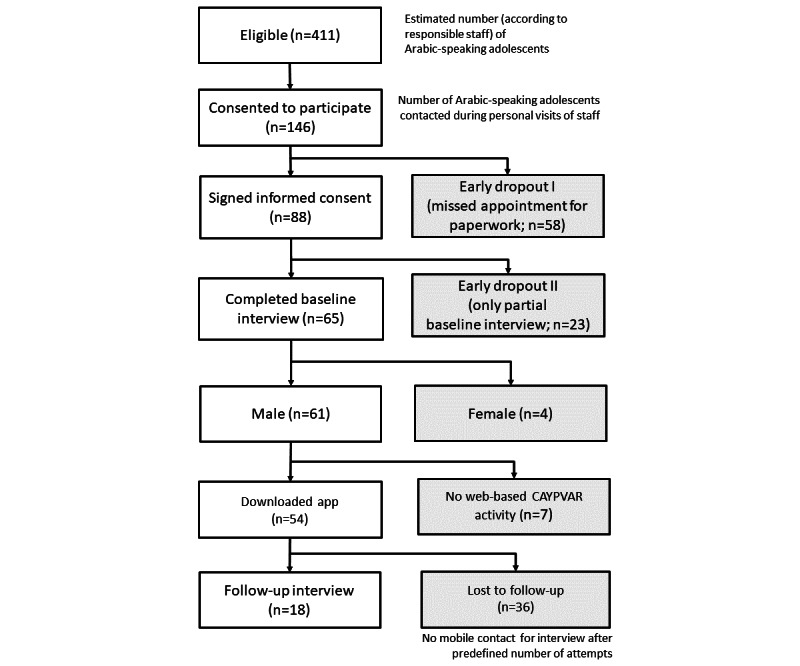
Consort.

The number of potentially eligible Arabic-speaking adolescents and young adults in the participating institutions was estimated by the facility staff to be 411 persons on the day of being contacted by our study team. The project team made personal recruitment visits and successfully asked 146 participants for their willingness to participate, but many of them (58/146, 39.7%) did not appear at the agreed appointment (Figure 2). Of the 88 participants who signed a written informed consent form, 23 (26.1%) participants stopped their baseline interviews early. Altogether, 65 baseline interviews were completed (male individuals: n=61, 94%; female individuals: n=4, 6%), in all cases with the help of either a professional interpreter (3 persons) or sometimes by a cohabitant speaking Arabic and German, good enough for translation. At follow-up, the interviews were mostly conducted via telephone to account for contact restrictions and relocation of the participants. Only the trained interpreters called the study participants in a priori fixed number (up to 8 times) and pattern (2 different daytimes) of attempts. For the following analyses, the responses of all participants who answered the respective items or interview parts were included, resulting in >61 responses as presented in Tables 2-4.

**Table 2:** Attitudes toward preventive behaviors and engagement in preventive behaviors at the baseline interview (n=65).

Preventive behavior	Attitude, n (%)^a^		Practiced behavior, n (%)^b^			
	Against	In favor	Never or rarely	Sometimes	Always	Not possible
...keep physical distance	4 (6)	61 (94)	4 (6)	19 (29)	31 (48)	11 (17)
...wear masks	3 (5)	62 (95)	1 (2)	13 (20)	43 (66)	8 (12)
...frequent hand washing	4 (6)	61 (94)	2 (3)	14 (22)	42 (65)	7 (11)
...frequent ventilating	4 (6)	61 (94)	5 (8)	12 (18)	39 (60)	9 (14)
...healthy food	4 (6)	61 (94)	4 (6)	22 (34)	31 (48)	8 (12)
...avoid mass gatherings	6 (9)	59 (91)	4 (6)	14 (22)	34 (52)	13 (20)
...contact-free greeting rituals	7 (11)	58 (89)	9 (14)	12 (18.5)	35 (54)	9 (14)
...sneezing into elbow	12 (18)	53 (82)	9 (14)	18 (28)	27 (42)	11 (17)
...mouth rinsing	15 (23)	50 (77)	15 (23)	12 (18)	30 (46)	8 (12)

^a^Attitudes toward preventive (“Since the beginning of the Corona pandemic, I think it makes sense to...”) were assessed on a 4-point scale (“applies not at all,” “applies rather not,” “rather applies,” “applies totally”). Scores were dichotomized for the analysis (ie, “against” and “in favor”).

^b^Actual practice of preventive behaviors (“Since Corona, I have gotten in the habit of...”) was assessed on a 5-point scale (“never,” “rarely,” “sometimes,” “always,” and “not possible in the housing facility”), whereas the first 2 categories (“never” and “rarely”) were summed up for the analysis.

**Table 3:** Self-assessed own knowledge about infectious diseases at the baseline (n=67) and follow-up (n=18) interviews.

Knowledge about infectious diseases	Self-assessment “yes”^a^	
	Baseline, n (%)	Follow-up, n (%)
I know the function of the immune system.	35 (52)	13 (72)
I understand what antibodies are.	24 (36)	6 (33)
I know the difference of viruses and bacteria.	29 (43)	13 (72)
I understand what viruses are.	50 (75)	13 (72)
I know the composition of human cells.	11 (16)	3 (17)
I know how antibodies work.	41 (61)	5 (28)
I understand what DNA is.	34 (51)	12 (67)
I know the difference of DNA and RNA.	12 (18)	7 (39)
I know the replication process of viruses.	16 (24)	6 (33)
I know about infectious diseases.	35 (52)	7 (39)

^a^One’s own knowledge of infectious diseases (“Please read each statement carefully and check how much the statement applies to you”) was assessed on a 3-point scale (“no,” “more or less,” “yes”).

**Table 4:** Knowledge of disease mechanisms and transmission paths at the baseline (n=67) and follow-up (n=18) interviews.

		Correct answer^a^	
		Baseline, n (%)	Follow-up, n (%)
**Knowledge of HIV**			
	Kissing^b^	23 (34)	7 (39)
	Hand shakes^b^	46 (69)	14 (78)
	Blood contact (eg, sex)^c^	61 (91)	15 (83)
	Only men susceptible^b^	37 (55)	15 (83)
**Knowledge of herpes**			
	Transmission by droplets^b^	27 (40)	7 (39)
	Sexual contact^c^	57 (85)	11 (61)
	Only shared drinking vessels^b^	23 (34)	13 (72)
	Only aerosols^b^	34 (51)	13 (72)
**Knowledge of COVID-19**			
	Droplets, aerosols, and smear^c^	63 (94)	17 (94)
	No transmission beyond 2-m distance^b^	25 (37)	8 (44)
	Exclusively droplets, smear infection^b^	5 (7)	0 (0)
	Only aerosols^b^	42 (63)	11 (61)

^a^One’s own knowledge of infectious diseases (“Please read each statement carefully and check what you think is the correct answer. How are HIV/Herpes/Corona viruses transmitted?”) was assessed on a 2-point scale (“true,” “not true”).

^b^The right answer was “not true.”

^c^The right answer was “true.”

### Characteristics of Study Participants

The sociodemographic and migration-related characteristics of the participants are shown in Table 5. At the baseline interview, participants were aged on average 24.3 (SD 4.5) years. Two screening instruments for participants’ mental health at baseline yielded hints for depressive disorder in 31% (19/61) of the participants (PHQ-4>6) [35] and for a “high tendency” to experience somatization (SSS-8>11 points) [36] in 62% (38/61) of the participants. The mean score for perceived self-efficacy [37] was relatively low in this sample. Most participants were of Syrian origin (34/61, 56%), followed by Yemen (12/61, 20%), and Iraq (5/61, 8%). The remaining participants were born in many different states of the Arabic-speaking world. Furthermore, 95% (58/61) of the participants stated Islam as their religious orientation, mostly Sunni Islam (52/61, 85%). Nearly 30% (18/61) had an education of not more than 9 years of school, whereas 56% (34/61) reported that they had visited secondary school for 13 years.

**Table 5:** Sociodemographic and migration-related characteristics (n=61).

Characteristics		Values
Age (years), mean (SD)		24.3 (4.5)
Education (years), mean (SD)		11.4 (2.2)
**Country of origin, n (%)**		
	Syria	34 (56)
	Yemen	12 (20)
	Iraq	5 (8)
	Somalia	3 (5)
	Eritrea	2 (3)
	Algeria	3 (5)
	Lebanon	1 (2)
	Palestine	1 (2)
**Religious faith, n (%)**		
	Islam	58 (95)
	Christian	1 (2)
	Other or none	2 (3)
Months since arrival in Germany, mean (SD)		7.1 (14.7)
Depressive symptoms, mean (SD)^a^		5.03 (3.8)
Somatic complaints, mean (SD)^b^		15.0 (6.7)
General Self-efficacy, mean (SD)^c^		29.6 (6.6)

^a^Assessed using Patient Health Questionnaire-4 [35].

^b^Assessed using Somatic Symptom Scale-8 [36].

^c^On the basis of General Self-efficacy Scale [41].

### Use of the CAYPVAR App

As shown in Table 6, there was a steep decrease in participation after baseline interviews. Of the 61 participants, only 54 (89%) participants downloaded the CAYPVAR app on their mobile phones, with a diminishing use tendency. A minority of less than 20% (12/61) of the participants watched the videos of week 3 to week 6. No participants were involved in winning the award of the photo competition. The frequency of playing the web-based 2020 game could not be determined, as this was an external link. A total of 43% (26/61) of participants clicked at least once on the link to that game.

**Table 6:** Use of information material in the app and participation in gaming elements (n=61).

			Participants’ involvement, n (%)
Downloaded the app			54 (89)
**Watched ≥1 video provided for the week**			
	Week 1	32 (52)	
	Week 2	16 (26)	
	Week 3	12 (20)	
	Week 4	11 (18)	
	Week 5	10 (16)	
	Week 6	11 (18)	
Games and incentives			32 (52)
Web-based game^a^			≥26 (≥43)
Participated in photo competition			0 (0)

^a^External link opened, but actual use could not be determined.

### Outcome of Major Study End Point I (Vaccination Readiness)

At the baseline interview, 77% (50/65) of participants had already been vaccinated against COVID-19. An additional 20% (13/65) of participants stated their willingness to get vaccinated as soon as possible. Only 5% (3/65) of participants were reluctant to be vaccinated. There were no indications of differing attitudes or behaviors between the different housing facilities.

### Outcome of Major Study End Point II (Knowledge of Disease Mechanisms)

With regard to preventive behaviors, participants were asked about their attitude toward and engagement in various behavioral measures to mitigate the spread of COVID-19 (Table 2). In the baseline interview, all these measures were extremely favored, and participants claimed to practice these measures “always” at least by 42% (27/65) of the sample. This was also true for 2 control measures with no or doubtful efficacy against transmission of the virus (ie, “healthy food” and “mouth rinsing”).

As statistical power was too low to test pretest-posttest differences between baseline and follow-up interviews (ie, only 15 participants were successfully matched instead of the 40 required for a power of 0.9), only descriptive values on attitudes toward and engagement in preventive behaviors among the follow-up interviewees could be captured as presented in Table 7. Attitudes favoring the behaviors were relatively lower, and no participant claimed to practice any of the behaviors “always.” The proportion of participants who rated themselves as hindered by their housing situation to practice the measures (column “not possible”) was relatively comparable between the baseline and follow-up interviews.

**Table 7:** Attitudes toward preventive behaviors and engagement in preventive behaviors at the follow-up interview (n=18).

Preventive behavior	Attitude, n (%)^a^		Practiced behavior, n (%)^b^			
	Against	In favor	Never or rarely	Sometimes	Always	Not possible
...keep physical distance	8 (44)	10 (56)	5 (28)	10 (56)	0 (0)	3 (17)
...wear masks	3 (17)	15 (83)	3 (17)	14 (78)	0 (0)	1 (6)
...frequent hand washing	6 (33)	12 (67)	2 (11)	14 (78)	0 (0)	2 (11)
...frequent ventilating	1 (6)	17 (94)	2 (11)	15 (83)	0 (0)	1 (6)
...healthy food	10 (56)	8 (44)	8 (44)	9 (50)	0 (0)	1 (6)
...avoid mass gatherings	7 (39)	11 (61)	7 (39)	8 (44)	0 (0)	3 (17)
...contact-free greeting rituals	2 (11)	16 (89)	3 (17)	12 (67)	0 (0)	3 (17)
...sneezing into elbow	5 (28)	13 (72)	2 (11)	15 (83)	0 (0)	3 (17)
...mouth rinsing	5 (28)	13 (72)	6 (33)	10 (56)	0 (0)	1 (6)

^a^Attitudes toward preventive (“Since the beginning of the Corona pandemic, I think it makes sense to...”) were assessed on a 4-point scale (“applies not at all,” “applies rather not,” “rather applies,” “applies totally”). Scores were dichotomized for the analysis (ie, “against” and “in favor”).

^b^Actual practice of preventive behaviors (“Since Corona, I have gotten in the habit of...”) was assessed on a 5-point scale (“never,” “rarely,” “sometimes,” “always,” and “not possible in the housing facility”), whereas the first 2 categories (“never” and “rarely”) were summed up for the analysis.

Concerning the possible selection bias of participants answering the follow-up interview, we found no indication of different scores with regard to mental health measures (depression, PHQ-4; somatization, SSS-8). Of the 18 participants who completed the follow-up interview, 15 (83%) participants declared that they had learned new facts about COVID-19 from the physician (KMA) while watching the video clips via the CAYPVAR app. A high proportion (16/18, 89%) of the participants expressed their trust in the physician and these new facts. Self-confidence in one’s own knowledge of disease mechanisms, although not statistically tested, tended to increase between baseline and follow-up interviews on a descriptive level with one exception: participants during the follow-up interview less often stated that they had knowledge about infectious diseases (Table 3).

In addition to confidence in one’s disease knowledge (Table 3), a knowledge test on potential transmission paths of 3 different infectious diseases (HIV, herpes, and COVID-19) yielded heterogeneous results as shown in Table 4. There was a high proportion of ignorance regarding all 3 infections, especially when transmission paths had to be excluded for a correct answer. For the question regarding COVID-19, the only correct answer was to include all 3 named pathways. Thus, the alternatives “solely via the air” and “via droplets and smear infections” were wrong. In sum, correctly identified transmission paths at baseline increased slightly from 6.8 (SD 2.6) answers to a mean value of 7.2 (SD 1.6) correct answers at the follow-up interview. For participants who answered the follow-up interview, a 2-tailed *t* test for dependent groups was performed, with t_15_=−0.60, which was not significant (*P*=.56).

## Discussion

### Principal Findings

#### Overview

The CAYPVAR study aimed to impart knowledge about COVID-19 and the available vaccines by implementing a mobile-based intervention with elements from serious games. This feasibility study yielded 2 main results. First, the vaccination rate or readiness among participants was very high at baseline. Second, an evaluation of the feasibility of our preventive intervention strategy could not be successfully achieved with regard to knowledge of disease mechanisms and attitudes toward preventive behaviors because of the small number of participants taking part in the follow-up interview. The enrollment of participants in this study was very difficult, and the dropout rate from the study among the enrolled participants was high.

#### Vaccination Rates

The high vaccination rate reported by the participants at baseline (50/65, 77%) is in contrast to the current literature. A review of general vaccine uptake in migrant populations in Europe showed that asylum seeker or refugee status increased the risk for undervaccination [42]. Acceptance of human papillomavirus, measles, and influenza vaccines was particularly low among Muslim migrants [42]. With regard to COVID-19, a qualitative interview study with recently arrived migrants and refugees in the United Kingdom reported that 72% of participants were hesitant to uptake a COVID-19 vaccine before the start of large-scale vaccination campaigns [43]. A recent French study reported a significantly lower COVID-19 vaccination rate among precariously housed and collectively accommodated migrants than among the general French population [44].

There are 2 possible explanations for the high vaccination rates observed in this study. First, participants might have perceived staff working in their collective housing facilities as reliable state representatives and information sources and thus followed their advice to get vaccinated. Tjaden et al [14] evaluated a Facebook campaign for COVID-19 vaccine information in a large sample of Arabic-, Turkish-, and Russian-speaking persons in Germany. In addition, they investigated the effect of the language and messenger (family, physician, government, or religious authority) of the advertisements. They showed that for Arabic-speaking participants, advertisements in Arabic led to more clicks on information pages and accesses to vaccination centers with web-based booking than those in German. In addition, a state representative as messenger of the advertisements was superior to religious leaders, physicians, or family as messengers.

Second, we conducted informal talks after the baseline interviews in this study. We learned that many participants feared a negative impact on their asylum proceedings if they had refused to participate in the vaccination campaigns organized by the staff of their collective housing facilities. A review of general vaccine uptake in migrant populations in Europe identified distrust in the health care system and fear of being questioned about one’s legal status as a barrier to accepting vaccination [42]. The opposite, that is, the fear of negative impacts on one’s legal status if not vaccinated, might have served as a facilitator of vaccination in this study. Nevertheless, the fact that most participants in this study had been vaccinated seems to have diminished their motivation to gain further knowledge on preventive measures against COVID-19 as presented in the CAYPVAR app.

#### Potential Reasons for Insufficient Feasibility

An evaluation of the feasibility of the presented intervention strategy could not be achieved because of the severe difficulties with enrollment in this study. Regarding recruitment, the following 2 obstacles may have hampered enrollment in this study. First, there was a large contrast between potentially eligible asylum seekers living in housing facilities and those who agreed to participate in the study. This could be explained by the limited presence of asylum seekers during the daytime in their housing institution during which the study team tried to make personal contact in the form of a recruitment visit. However, the striking difference between the individuals who stated their interest in participating in the study (n=146) and those who signed the informed consent form (n=88) cannot be explained by this. In several cases, the participants were relocated to another institution during the week following the recruitment visit as reported by the housing staff. However, it also seemed that agreeing to the invitation to participate in the study during the first recruitment visit might not have been sufficient to appear at the agreed appointment a few days later. It can be speculated that refugees’ “lifes on hold” provoked “disintegration of time” that already in 2010 has been described in a Swedish study [45]. Early dropout during the baseline interview (n=23) in this study might be attributable to mistrust toward the study team, related to the fear of a possible negative impact on their asylum procedure. To counter such problems, we changed the enrollment procedure by conducting every baseline interview immediately after the first encounter with a potential participant instead of a separate recruitment visit beforehand.

Second, perhaps the participants in this study did not feel a “need for cognition” about COVID-19 because most of them had already been vaccinated and therefore had also undergone a consultation with the vaccinating physician. This study showed the young refugees’ limited general and COVID-19–related factual knowledge of infectious diseases and the transmission paths. For example, 30% (20/67) of participants stated that HIV could be transmitted via handshakes, and 36% (24/67) claimed that COVID-19 was only transmitted via aerosols. This finding is in line with other studies on refugees’ knowledge [8-10]. Kananian et al [8], for instance, reported that Arabic- and Farsi-speaking adult refugees in Germany had less knowledge about COVID-19 than matched nonrefugee participants. In addition, the fact that most participants in this study had been vaccinated could also mean that participants have felt “unsusceptible” owing to the vaccination and thus did not see the need to learn more about the virus and pandemic through CAYPVAR app.

Moreover, continued participation in this study was low, leading to a high number of dropouts during the intervention period and at the follow-up assessment. Drawing on the research on psychological interventions for refugees with mental health problems, further potential explanations for the low adherence of participants can be found. First, prominent postmigration stressors of asylum seekers are overcrowded and inadequate housing conditions or the prolonged or uncertain asylum process [46]. A review of contextual factors on the mental health outcomes of asylum seekers in Germany has identified, among other factors, living in a shared accommodation, poor language skills, and an uncertain asylum status as risk factors for psychological symptoms [47]. In this study, all participants were waiting for their asylum request to be processed (95%) or appealed against a rejected asylum request (5%). Psychological symptoms in asylum seekers, in turn, are associated with various difficulties, including communication and learning problems (eg, difficulties learning German or finding employment) [48]. Thirty-one percent of participants in this study reported clinically relevant depressive symptoms, and 62% experienced increased somatic symptoms. As in other studies on the mental health of refugees, the resulting concentration problems and therefore difficulties focusing on the video clips or short quizzes presented in the CAYPVAR app seem plausible.

Second, the participants in this study had settled in Germany only recently, on average, 7 months ago. One could speculate that the problems of these newly settled asylum seekers (ie, unclear residence status, precarious and temporary housing conditions, and no mobile phone contacts or stable Wi-Fi access) might have constituted specific barriers not only to enrollment but also to continued participation. Thus, the current intervention strategy might not have been feasible for the specific group under investigation, but this does not necessarily apply to other groups of asylum seekers and refugees. For example, the initial settlement period (eg, long waiting periods for an asylum decision, collective housing conditions, and short-term relocations) has been described as an additional source of stress for asylum seekers [49]. Therefore, it has been proposed to assess refugees’ mental health after their arrival and again after the initial resettlement period [49]. It seems plausible that refugees who had been in Germany for a longer time and had more stable living conditions (eg, having been granted residence permits and allowance to seek employment) would have responded differently to the intervention strategy of this study.

Third, the dropout rate was extremely high in this study despite the precautions taken such as age-adapted information materials (ie, video clips) and incentives in the form of quizzes and competitions. In addition, we established a cultural match between participants and assessors (DS) and persons shown in the video clips (DS and KMA) and used culture-sensitive modes of delivery of the intervention such as simple language [31,32]. Moreover, we used a medium degree of guidance by providing individualized feedback on the quizzes after each video clip and personal contact with the study team for the baseline and follow-up interviews. This was based on the evidence that digital interventions with at least a minimal degree of personal contact yielded greater symptom reductions than unguided interventions in adult nonrefugees with depression [50]. However, the high dropout rate in this study corresponds to that reported by Lindegaard et al [51], who evaluated a smartphone-based cognitive-behavioral intervention for young Farsi-speaking refugees with symptoms of mental disorders. They reported a dropout rate of 80% and were thus unable to evaluate the potential efficacy, concluding that their intervention was not feasible. The most important barriers to continued participation were the lack of human contact and symptoms such as concentration problems.

### Conclusions

In conclusion, the concept of this study aimed at developing and evaluating the feasibility of a primary preventive intervention against COVID-19. The design of the study did not allow us to unambiguously decide which or which exact combination of the observed obstacles was pivotal for the low feasibility. However, a combination of psychological and structural factors should be considered: a combination of sample characteristics (eg, low need for cognition), external barriers (eg, living conditions allowing no retreats to watch the videos undisturbed), and a dynamic pandemic situation with rapidly changing legal regulations as a new virus variant became dominant could have fostered low involvement in this study. Owing to difficulties with enrollment and continued participation, mobile app–based infotainment for providing information (ie, the CAYPVAR app) could not be applied to an extent that could be regarded as “minimum dose” for achieving behavioral effects, even if we had a more lenient design [52]. Establishing continuously updated platforms in the first language that offer health-related tailored “infotainment” plus other practical information about the host country might be a more promising approach for the target group [53].

In addition, some of the obstacles encountered were beyond the scope of the study design such as the speed of the spread of new variants or rapidly changing legal regulations. In addition, the precarious and unstable living conditions of our target group, recently arrived young asylum seekers, could not be changed. Therefore, we do not recommend optimizing the present intervention strategy for this group, but we do not rule out its potential feasibility for refugees with more stable living conditions.

Thus, we doubt the usefulness of a preventive mobile app–based intervention concept in the target group. Possibly, improvements would be achieved if the focus shifted from behavioral (even primary) prevention to primordial prevention [54]. A simple illustration of this argument can be seen when the size of the housing institution is <10 persons, as compared with collective housing with several hundred inhabitants. By housing people in smaller institutions, the primary prevention technique of contact reduction is unobtrusively enforced, without any educational campaign and any formal “no-contact rule” for the inhabitants. If preventive measures are considered from an organizational perspective [55], this could open a more promising way of mitigating the spread of disease in the target group.

## Abbreviations

CAYPVAR: COVID apps for young adults for preventing transmission and promoting vaccination among refugees
PHQ-4: Patient Health Questionnaire-4
SSS-8: Somatic Symptom Scale-8
WLAN: wireless local area network

## Data Availability

The data sets generated and analyzed during this study are available from the corresponding author on reasonable request.
